# Are neural crest stem cells the missing link between hematopoietic and neurogenic niches?

**DOI:** 10.3389/fncel.2015.00218

**Published:** 2015-06-17

**Authors:** Cécile Coste, Virginie Neirinckx, André Gothot, Sabine Wislet, Bernard Rogister

**Affiliations:** ^1^Groupe Interdisciplinaire de Génoprotéomique Appliquée-Neurosciences, Unit of Nervous System Disorders and Treatment, University of LiègeLiège, Belgium; ^2^Groupe Interdisciplinaire de Génoprotéomique Appliquée-Cardiovascular Sciences, University of LiègeLiège, Belgium; ^3^Hematology Department, University HospitalLiège, Belgium; ^4^Groupe Interdisciplinaire de Génoprotéomique Appliquée-Development, Stem Cells and Regenerative Medicine, University of LiègeLiège, Belgium; ^5^Neurology Department, University HospitalLiège, Belgium

**Keywords:** hematopoietic stem cell, niche, neural crest stem cell, neural stem cell, signaling pathways

## Abstract

Hematopoietic niches are defined as cellular and molecular microenvironments that regulate hematopoietic stem cell (HSC) function together with stem cell autonomous mechanisms. Many different cell types have been characterized as contributors to the formation of HSC niches, such as osteoblasts, endothelial cells, Schwann cells, and mesenchymal progenitors. These mesenchymal progenitors have themselves been classified as CXC chemokine ligand (CXCL) 12-abundant reticular (CAR) cells, stem cell factor expressing cells, or nestin-positive mesenchymal stem cells (MSCs), which have been recently identified as neural crest-derived cells (NCSCs). Together, these cells are spatially associated with HSCs and believed to provide appropriate microenvironments for HSC self-renewal, differentiation, mobilization and hibernation both by cell-cell contact and soluble factors. Interestingly, it appears that regulatory pathways governing the hematopoietic niche homeostasis are operating in the neurogenic niche as well. Therefore, this review paper aims to compare both the regulation of hematopoietic and neurogenic niches, in order to highlight the role of NCSCs and nervous system components in the development and the regulation of the hematopoietic system.

## Introduction: adult stem cells niches in the adult bone marrow and brain

Stem cells are characterized by their continuous self-renewal ability and pluri- or multipotentiality, and could consequently give rise to a wide panel of cell types. Non-germinal stem cells are classified into different categories. Embryonic stem cells (ES) are found in the inner cell mass of the blastocyst and are pluripotent stem cells that generate any mature cell of each of the three germ layers. Somatic stem cells are tissue-specific and more restricted than ES cells in terms of fate choice and of differentiation capabilities. They can be isolated from various fetal and adult tissues, and therefore constitute an attractive supply of material for cell therapy.

Stem cell niches were deeply analyzed over these last years in order to better understand and control stem cell proliferation and differentiation. Indeed, the concept of niche refers to a microenvironment harboring stem cells, which regulates both their self-renewal property and cell fate choice. During embryonic development, various factors inside the niche act on stem cells and modify gene expression to induce their proliferation or differentiation, in order to favor the development of the fetus.

**Figure 1:**
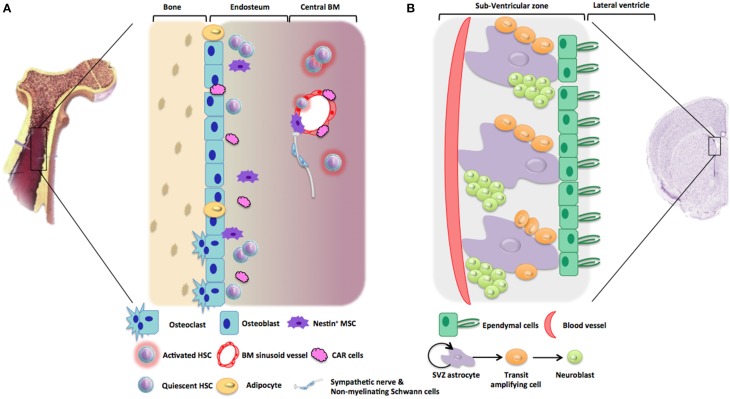
**Representative architecture of hematopoietic and neurogenic niches. (A)** Hematopoietic niches are microenvironments into bone marrow stroma which support HSC quiescence and self-renewal. Endosteal niche is mainly composed of osteoblasts, osteoclasts, adipocytes, CAR cells, stromal cells, nestin^+^ MSC, altogether maintaining HSC in a quiescent state. In comparison, vascular niche located near to BM sinusoid vessels is composed of perivascular (nestin^+^) stromal cells and CAR cells favoring HSC activation and recruitment. Non-myelinating Schwann cells were also described to be involved in HSC maintenance. **(B)** The subventricular zone (SVZ) is one of the neurogenic niches in the adult brain. This neurogenic niche is in contact with ependymal cells that line the cerebrospinal fluid (CSF) circulating in the lateral ventricles. Neural stem cells (NSC) are localized in contact with this ependymal cell layer and with blood vessels. Type B cells or SVZ astrocytes proliferates thanks to asymmetric division giving rise to type C cells, or transit amplifying cells, which will further differentiate into type A cells or neuroblasts. During adult neurogenesis, these neuroblasts will proliferate and migrate toward the olfactory bulb or the striatum.

Within the adult human body, the main role of those niches is the maintenance of stem cell quiescence. Mammalian adult stem cell niches have been described in many tissues including the testis, the hematopoietic tissue, the skin, the intestine, or the brain. Several important factors regulate stem cell characteristics within the niche, such as adhesion molecules that mediate important cell-cell interactions between stem cells and supportive cells, neighboring differentiated cells or matrix components. In some cases of tissue injury, the surrounding environment acts on the niche and actively recruits stem cells to either self-renew or differentiate, to generate new cells and tissues. In the following paragraphs, we will more precisely focus on hematopoietic stem cell and neural stem cell niches (Figure 1).

The concept of hematopoietic niche was first introduced in 1978, when Schofield and collaborators observed that surrounding bone marrow stromal cells strongly supported hematopoietic stem cells (HSCs) maintenance and activity in an *in vitro* co-culture system, while spleen cells were less efficient in insuring HSC regulation (Schofield, 1978). According to Schofield and others, the HSC niche can be defined as an heterogeneous microenvironment inside the trabecular bone cavity, which is composed of specialized cell populations that play essential(s) role(s) in regulating the self-renewal and differentiation of HSC through both surface-bound factors and soluble signals, together with mature progeny released into the vascular system (Uccelli et al., 2008; Renstrom et al., 2010). Two functional subdivisions of HSC niches are described in the adult bone marrow (BM): (1) the endosteal niche is composed inter alia by osteoblasts lining the endosteum (Nilsson et al., 2001; Calvi et al., 2003; Zhang et al., 2003) and regulates HSC's quiescence by maintaining them in G0/G1 phase (Emerson, 2007); whereas (2) vascular niches host HSCs in close relationships with vascular endothelium of marrow sinuses and mostly embraces HSC proliferation, differentiation, and recruitment (Kiel et al., 2005; Kiel and Morrison, 2008). Maintenance of the stem cell pool and formation of differentiated progenitors are therefore harmonized in order to achieve a steady-state hematopoiesis.

Even if the cellular composition of HSC niches still remains elusive at some points, mesenchymal stem cells (MSCs) of the BM stroma are well-known cellular components of the HSC niche which regulate hematopoietic processes through the secretion of many growth factors and cytokines (see below) (Anthony and Link, 2014). In addition, *in vivo* reconstitution of the hematopoietic niche may be achieved upon transplantation of MSCs or of a subpopulation of osteoprogenitors, which tightly interact with sinusoids and secrete growth factors (Caplan, 1991; Muguruma et al., 2006; Sacchetti et al., 2007). Many studies also demonstrated the implication of perivascular cells (Crisan et al., 2008; Ramasamy et al., 2014) in the regulation of hematopoiesis. Interestingly, Méndez-Ferrer and collaborators recently shown that nestin^+^ MSCs are essential components of the endosteal niche and are required for the proper regulation of hematopoietic processes (see below) (Mendez-Ferrer et al., 2010; Isern et al., 2014). More recently, they demonstrated that those nestin^+^ MSCs were neural crest-derived stem cells (Isern et al., 2014), which are known to persist in the adult bone marrow and in various other adult tissues such as the skin or the dental pulp (Nagoshi et al., 2008; Achilleos and Trainor, 2012). Together with the identification of non-myelinating Schwann cells inside the bone marrow (Yamazaki et al., 2011), those findings highlight the contribution of nervous system elements (and more particularly the neural crest) to the formation and maintenance of the hematopoietic system.

As first demonstrated in the late 90's (Eriksson et al., 1998; Doetsch et al., 1999; Gage, 2000), the adult nervous system also shelters specific microenvironments that both support the maintenance of neural stem cells (NSCs) alongside with the generation of newborn cells, mostly neurons in adulthood (Zhao et al., 2008). Neurogenic sites are located within (1) the subventricular zone (SVZ) along the wall of lateral ventricles, where NSCs give rise to neurons migrating in the olfactory bulb and the striatum (Ernst et al., 2014), and (2) in the hippocampal subgranular zone, where NSC-derived neurons integrate the *dentatus gyrus*. NSC maintenance and neurogenesis are well-regulated by numerous signals provided by the local blood vessels network with highly specialized properties (Shen et al., 2004; Tavazoie et al., 2008), the cerebrospinal fluid that circulates along the ventricles (Silva-Vargas et al., 2013), and by the surrounding cells (Tavazoie et al., 2008).

Although NSC niches are central nervous system (CNS) structures that are not supposed to hold neural crest-derived cells, it appears that many similarities and connections between HSC and NSC niches could be revealed by the recent literature and presented in Figure 1. As mentioned before, this review aims to compare what is known about the mechanisms that regulate both hematopoietic and neurogenic events, with a focus on the potential roles of neuroectodermal-derived cells (NCSC in hematopoiesis and NSC in neuropoiesis) in the orchestration and the regulation of the adult stem cell niches.

## Cellular and molecular regulation of hematopoietic and neurogenic processes

**Figure 2:**
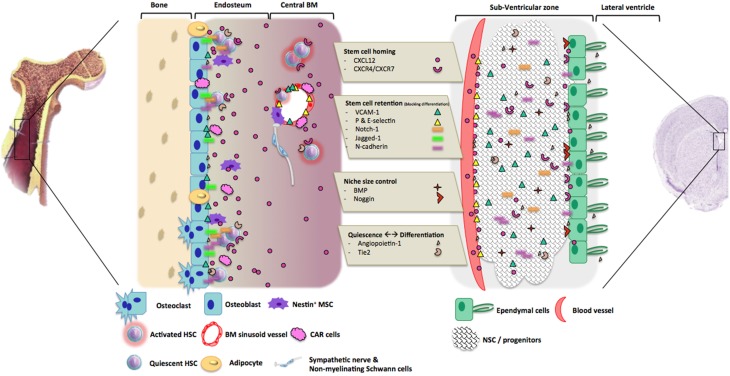
**Molecular processes involved in hematopoietic and neurogenic niches regulation**. The molecules that are involved in the regulation of hematopoiesis and neurogenesis could be divided into three main groups, according to their roles in niches. 1. Stem cell homing: the couple CXCL12/CRCR4-7 are the most important component as they allow HSC or NSC homing and stemness into the niche. 2. Stem cell retention. After stem cells recruitment and homing, adhesion molecules and their ligands are involved in stem cells retention by blocking their differentiation and migration. 3. Control of niche size: BMP signaling pathway regulates niches size through the promotion of stem cells differentiation and or mobilization. 4. Control of quiescence and differentiation in HSC and NCSC niches: Angiopoietin-1 and its receptor Tie2 play different roles within the two niches from HSC quiescence maintenance to NSC proliferation and differentiation. Other actors could be involved, like sympathetic neurons regulating HSC attraction and mobilization into the blood flow.

As mentioned before, adult stem cell niches have been described in many different tissues. Despite significant anatomical differences, those tissues share many common features concerning the extracellular mechanisms by which the stem cell population is regulated within the niche (Zapata et al., 2012). We therefore decided to compare hematopoietic and neural stem cell niches, and to have a look on their regulation pathways (Figure 2).

### CXCL12: the most important cytokine signalization for stem cell homing and maintenance

CXCL12 (also called stromal-derived factor 1 or SDF-1) is a member of the chemoattractive cytokine family (chemokines), and is essential for the proper proceedings of hematopoiesis, general ontogeny, cardiovascular formation, and neurogenesis. Indeed, it has been observed that CXCR4-deficient mice (lacking the receptor for CXCL12) die around birth and present important defects in hematopoietic and nervous system, such as a reduced myelopoiesis and B-lymphopoiesis, and impaired neuronal migration in the cerebellum (Ma et al., 1998).

During bone marrow ontogeny, colonization of the bone marrow (BM) involves recruitment and engraftment of circulating myeloid cells and HSCs originating from the fetal liver, which will then interact with BM endothelium in order to migrate toward endosteal or vascular niches (Ara et al., 2003). This capture step is mainly driven by the secretion of CXCL12 by CXCL12-abundant reticular (CAR) cells (a subset of perivascular stromal cells), which acts on CXCR4 receptor at the surface of HSCs (Sugiyama et al., 2006). More recently, Isern and collaborators demonstrated that the capture step was also regulated by the presence of post-migratory NCSCs located in the BM niche. Those NCSCs secrete CXCL12 and attract HSCs that colonize the BM tissue in newborn mice (Isern et al., 2014).

Throughout adult life, maintenance of HSC quiescence, survival and self-renewal in the adult BM niche also relies on CXCL12/CXCR4 signalization by nestin^+^ mesenchymal stem cells, CAR cells, osteoblasts, and endothelial cells, which differentially regulate the niche homeostasis (Greenbaum et al., 2013). Noteworthy, CXCL12-CXCR4 axis seems to be conserved during the evolution. Indeed, it was recently reported that zebrafish HSC homing in BM perivascular niche is dependent of CXCL12-expressing fibroblastoïd stromal cells, homologous of the CAR cells (Tamplin et al., 2015). Importantly, although CXCR4 is the most described receptor for CXCL12, it appears that another receptor (namely CXCR7) also has important role in HSC regulation and dysfunction (Melo Rde et al., 2014; Torossian et al., 2014).

In comparison, in the developing brain, CXCL12-CXCR4 axis regulates the migration of neuronal precursors in the cerebellum (Vilz et al., 2005), the dentate gyrus (Bagri et al., 2002; Kolodziej et al., 2008), the cerebral cortex (Stumm et al., 2003), the dorsal root ganglia (Belmadani et al., 2005) and some nuclei in the brainstem and hypothalamus (Schwarting et al., 2006). The other receptor for CXCL12, CXCR7 (Sanchez-Martin et al., 2013), also seems to be involved in CXCL12 signalization during brain ontogeny and homeostasis (Schonemeier et al., 2008). In the adult brain, CXCL12 and its receptors are expressed by a lot of different neuronal populations located in the cortex, the mesencephalon or the hypothalamus (Banisadr et al., 2003). This chemokine is also secreted by ependymal cells and endothelial cells of the SVZ (Kokovay et al., 2010; Goffart et al., 2015), which both form a vascular neurogenic niche and contribute to the maintenance of stemness/migration in the adult brain (Shen et al., 2008).

### VCAM1 and N-cadherin: homing and balance between stem cell retention and migration

Homing of HSCs during development into BM also involves cell-cell interactions. Those are mediated by adhesion molecules expressed by BM sinusoidal endothelial cells and stromal cells (Simmons et al., 1992), such as vascular cell adhesion molecule 1 (VCAM1), P-selectin and E-selectin (which respectively attract circulating HSCs by acting on α4β1 integrin, CD162 and E-selectin ligands) (Frenette et al., 1998). The expression of VCAM1 on those cells is also responsible for the regulation of normal cell trafficking between the BM and the blood stream in adult individuals (Ulyanova et al., 2005). Of note, additional ligands of α4β1 integrin, namely osteopontin and fibronectin, are also involved in maintaining HSCs in a quiescent state (Jiang et al., 2000; Nilsson et al., 2001; Stier et al., 2005). Similarly, VCAM1 is expressed by neural precursors in the adult brain SVZ and largely contributes to the niche architecture and function. Indeed, it appears that VCAM1 maintains NSC in a stem cell state by inducing the formation of reactive oxygen species (Le Belle et al., 2011). Just as in the adult BM, VCAM1 acts as a sensor and modulates stem cell maintenance/migration in response to environmental signals (Kokovay et al., 2012).

In the developing bone marrow as well as in the adult hematopoietic tissue, homotypic N-cadherin-mediated cell interactions between spindle-shaped N-cadherin expressing osteoblasts (SNOs) and HSCs are critical for regulating stem cell engraftment and quiescence, in the endosteal niche (Zhang et al., 2003). However, since KO mice for N-cadherin do not develop further than mid-gestation (Radice et al., 1997), there is therefore no functional evidence for N-cadherin role in the bone marrow. Even though, other molecular pathways also contribute to stem cell retention (Kiel et al., 2007). As an example, angiopoietin-1-dependent regulation of N-cadherin increases HSC adhesion within the endosteal niche (Arai et al., 2004). During cerebral cortical development, N-cadherin-mediated interactions between precursors within the ventricular zone coordinate signaling pathways that regulate proliferation and differentiation. N-cadherin-dependent cell contact regulates β-catenin signaling though Akt activation, and precursors thus regulate their own differentiation, survival and migration (Zhang et al., 2010, 2013).

N-cadherin also mediates NSC anchorage to ependymal cells and quiescence within the SVZ, while suppression of N-cadherin function promotes NSC migration and differentiation (Yagita et al., 2009). This interaction is regulated by membrane-type 5 metalloproteinase (MT5-MMP), which dynamically modulate the proliferative status of NSCs through cleavage of N-cadherin adhesive contacts (Porlan et al., 2014). In pathological conditions, N-cadherin interactions could also be disrupted by ADAM10, which induces cytoskeletal rearrangements in NSC and migration from the SVZ toward demyelinated lesions (Klingener et al., 2014).

### Angiopoietin-1: from quiescence to differentiation

Angiopoietin-1 is an endothelial growth factor that is critical for division, survival, and adhesion of endothelial cells, via its tyrosine kinase receptor Tie-2 (Suri et al., 1996). Within the endosteal niche, HSCs are maintained in a quiescent state thanks to the secretion of angiopoietin-1 by osteoblasts, acting on Tie-2 receptor at the surface of HSCs (Arai et al., 2004).

In the adult brain, perivascular astrocytes, endothelial cells, ependymal cells, and choroid plexus are sources of angiopoietin-1. On the other hand, Tie-2 is express by non-endothelial cells, especially in neurons and stem cells from human and mouse brain, but also in glia (and glioblastoma cells (Rosa et al., 2010). *In vitro* studies show that angiopoietin-1 has pro-neurogenic effect through Tie-2 activation, and promote neurite outgrowth and synaptogenesis in sensory neurons (Kosacka et al., 2005, 2006). Angiopoietin-1 stimulates adult SVZ-derived NSC proliferation *in vitro*, and also increases differentiation in functional neurons and axonogenesis (Rosa et al., 2010). Angiopoetin-2 (another member of angiopoietin growth factors) also acts on Tie2 receptor and promotes NSC differentiation into neuronal lineage, and regulates neural progenitor cell migration through MMPs activity (in a Tie2-independent manner) (Liu et al., 2009).

### BMP signaling pathway: controlling niche size and stem cell differentiation

Bone morphogenic proteins (BMPs) are members of the transforming growth factor β family. Among them, BMP4 signaling regulates mesoderm cell commitment into HSC and differentiated myeloid cells during embryogenesis and hematopoietic tissue development (Chadwick et al., 2003; Durand et al., 2007) (reviewed in Sadlon et al., 2004). Moreover, BMP4 is expressed in osteoblasts, endothelial cells, and megakaryocytes (Goldman et al., 2009), and is involved in bone marrow niche homeostasis in adulthood by controlling HSC number and preserve niche size (by signaling through BMP receptor type IA) (Zhang et al., 2003). Interestingly, it appears that SMAD-dependent BMP signaling also regulates CXCL12 secretion in the BM niche, then influencing homing, engraftment, and mobilization of HSCs (Khurana et al., 2014).

In the developing brain, BMPs induce astroglial and neuronal differentiation of NSCs and precursors in the embryonic SVZ and developing cortex (Gross et al., 1996; Li et al., 1998), and inhibit neurogenesis (Shou et al., 1999).

BMPs are also expressed in the adult SVZ where they prevent neuroblast production from precursors by directing them into a glial lineage. However, neurogenic environment is maintained by ependymal cells secreting Noggin, which inhibits BMP signaling in the SVZ and stimulates neurogenesis (Lim et al., 2000).

### Notch signaling pathway: role in expansion of undifferentiated stem cells

Notch signaling plays fundamental role in embryogenesis by mediating cell proliferation, cell differentiation and cell fate decision (Artavanis-Tsakonas et al., 1999). A Notch-mediated crosstalk takes place in the BM niche, wherein Notch-1 is expressed by HSCs (and by other mature blood cell types) (Milner et al., 1994) and its ligand Jagged-1 is expressed by osteoblasts. The expression of Jagged-1 by the endosteal niche cells is stimulated by the parathyroid hormone (Calvi et al., 2003). Interestingly, Jagged-1 expression could also be identified in NCSC from adult mouse bone marrow, using a micro-array approach (GSE30419) (Wislet-Gendebien et al., 2012). Notch-1 signalization enhances stem cell renewal, but also favors lymphoid lineage and particularly T-cell differentiation (Bigas and Espinosa, 2012).

Similarly, in the adult SVZ, Notch signaling plays a role in the maintenance of stem cell population, and inactivation of the pathway depletes NSC pool and induces neuronal differentiation. More precisely, in human developmental neocortex, Notch signaling maintains a pool of progenitor cells called non-ventricular radial glia-like cells, which are able to differentiate into neurons (Hansen et al., 2010). This mechanism is regarded as a critical evolution step allowing the increase of neuron number in human telencephalon. Moreover, it was also reported that Notch actively cooperates with the pathway triggered by the EGF-receptor to balance the neural stem cells population with the neuronal precursor population in the adult SVZ (Aguirre et al., 2010).

### Nervous system regulates stem cells homing and exit from their niche

Both adult hematopoietic and neurogenic regions depend critically on nervous system signals. Indeed, sympathetic noradrenergic neurons regulate the attraction of HSCs to their niche, and their mobilization into the blood flow, in cooperation with G-CSF (Katayama et al., 2006). Furthermore, it appears that a denervation of autonomic nerves in the BM leads to a reduced number of non-myelinating Schwann cells (contributing to HSCs maintenance through TGFβ signaling) (Yamazaki et al., 2011). As already mentioned, these non-myelinating Schwann cells have close similarities with NCSCs.

Neuronal afferences contacting the adult SVZ are also known to regulate many parameters of the niche, according to the neurotransmitters that are secreted (reviewed in Young et al., 2011). Neurogenesis is therefore impaired in pathological conditions such as Parkinson's disease, when afferences from the striatum are lost (L'Episcopo et al., 2012).

## Could neural crest stem cells from HSC niches explain the similarities between hematopoietic and neurogenic niche signals?

During development, neural crest cells (NCCs) constitute a transient population of multipotent cells that arise at the border of the neural plate. After induction, NCCs delaminate, undergo epithelial-to-mesenchymal transition and migrate in discrete streams (cardiac, trunk, cranial, vagal NCCs) toward different tissues, finally giving rise to neurons and glia of the peripheral nervous system, melanocytes, craniofacial osteocytes, chondrocytes, etc. (Achilleos and Trainor, 2012; Mayor and Theveneau, 2013). Beside a well-determined transcriptional regulation (Anderson, 1994; Hong and Saint-Jeannet, 2005), numerous extracellular signals, growth factors, and adhesion molecules finely regulate different parts of this sequence.

### CXCL12/CXCR4 axis

The role of CXCL12/CXCR4 signalization axis in the migration of neural crest cells during development is well-defined. CXCL12/CXCR4 (and not CXCR7) chemoattractant signaling is required for the proper progression and migration of cardiac neural crest cells (NCCs) toward their appropriate locations in the developing heart (Escot et al., 2013) as well as for the correct development of craniofacial/orofacial cartilages that result from cranial NCCs migration (Olesnicky Killian et al., 2009; Rezzoug et al., 2011). This signaling axis is also required for the migration of sensory neurons and DRG formation (Belmadani et al., 2005). Overall, data of the literature underlines the importance of CXCL12/CXCR4 signaling during NCC migration. Still, it appears that NCCs rather respond to environmental secretion of CXCL12 instead of producing it themselves, as it is the case for adult bone marrow NCSCs (Isern et al., 2014).

### Adhesion molecules—VCAM-1 and N-cadherin

Migratory NCCs progress along defined pathways and cell adhesion molecules are required to allow NCC interactions with each other and with environing tissues (reviewed in McKeown et al., 2013). They express the α4β1 integrin enabling them to respond to a VCAM1 stimulus (Testaz et al., 1999). However, the same study showed that the NCCs migration cannot be triggered by only VCAM1/α4β1 integrin interaction, but also requires also a fibronectin stimulus. NCC emigration from the neural tube is also mediated by N-cadherin, which is highly expressed in premigratory NCCs and then switched off in favor of weaker type II cadherins (Mayor and Theveneau, 2013). Indeed, its overexpression disrupts the proper cell migration pattern of NCCs (Nakagawa and Takeichi, 1998).

### BMPs

Together with Wnt signaling, BMPs are important for the induction of neural crest in the earliest embryonic developmental steps (Raible, 2006). This important role of BMPs in NCC specification is also exemplified by the fact that NSCs put in culture and treated with BMP2 are induced to a neural crest fate and choroid plexus mesenchyme, after an epithelial-to-mesenchymal transition. The cells then differentiate into smooth muscle cells and peripheral nervous system glia (Sailer et al., 2005). Later during development, BMP2/4/7 derived from the wall of the dorsal aorta and surrounding mesenchyme induce NCCs to become precursors of sympathetic neurons and chromaffin cells, so-called sympatho-adrenal progenitors. They can be identified by their expression of distinct sets of transcription factors, most notably Phox2B, and components of the catecholaminergic synthetic machinery, as, e.g., tyrosine hydroxylase and dopamine ß-hydroxylase (reviewed in Unsicker et al., 2013). The inducing activity of BMPs in catecholaminergic neurons is also consolidated by the observation that BMPs are also able to stimulate NCC differentiation in enteric dopaminergic neurons (Chalazonitis and Kessler, 2012). This importance of BMPs in sympathetic and/or catecholaminergic neuron progenitor differentiation could also be involved in bone marrow regarding the implication of sympathetic innervation in the HSC niches (see above).

### Notch

Notch was recently suspected to play a role in the NCCs differentiation. In self-renewing pre-migratory NCCs induced from human pluripotent stem cells, Noisa et al. observed that Notch increases the expression of neural-crest-specifier genes (*SLUG* or *SNAIL2, SOX10*, and *TWIST1*) (Noisa et al., 2014) Moreover, Notch is then a brake of NCCs migration and the inhibition of Notch signaling is followed by a neuronal differentiation of these cells. Using *in vitro* and *in vivo* models, Morisson et al. demonstrated that Notch inhibits NCCs neuronal differentiation and activates the glial fate, mainly the Schwann cell phenotype (Morrison et al., 2000a,b) but not the satellite cells, the teloglia of somatic motor nerve terminals or the enteric glia (reviewed in Kipanyula et al., 2014).

## Conclusions

In light of this review, it appears that the relationship between hematopoietic and nervous systems, at least at the molecular level, has been under-estimated for many years. The main reason probably resides in the fact that the hematopoietic system has been well-described as a highly regenerating system for many years, while the nervous system is the ultimate example of a non-, or at least poorly-, regenerating system. However, the description of neurogenic niche regulation in the adult mammalian brain (including in humans) and the recent findings concerning several regulatory cell components of hematopoietic niches together shed the light on the obvious similarities concerning the molecular regulation pathways of the two systems. Moreover, increasing description of the nervous regulation of hematopoietic function, together with the putative importance of the relationship between SVZ vasculature and NSCs, seems to be the dawn of an interpenetration of both systems. Likewise, recent findings demonstrating that crayfish neurons are generated from the immune system is another example of this crisscross (Benton et al., 2014). We therefore suggest that in mammals, the interpenetration of both systems relies, at least partly, on neural crest derivatives present in bone marrow. A better knowledge of the properties and the roles of these cells could shed a light on the hematopoietic niche regulation, but could also feed new hypotheses for exploring and understanding neural stem cell niches of the adult brain.

Finally, this review should deliver another important message concerning the common regulation modes of both systems and their possible common dysregulations in pathological conditions (e.g., in leukemias and gliomas). Indeed, in both systems, adult niches have been demonstrated to provide a sanctuary for subpopulations of leukemic, but also glioblastoma cells that escape chemotherapy- and/or-radiotherapy induced death. Indeed, xenografted glioblastoma cells were recently shown to migrate toward the SVZ upon stimulation by CXCL12, which is secreted by endothelial cells (Goffart et al., 2015). It appears that this NSC niche also constitutes a particular microenvironment that promote glioblastoma cell maintenance (Goffart et al., 2013). On the other hand, the hematopoietic niche is also a key environmental regulator of leukemia stem cell proliferation, survival, and migration (Tabe and Konopleva, 2014). In both cases, cancer stem cells seem to share important features with stem cells located in the niche and are likely to be equally influenced by these specific microenvironments. Further understanding about the molecular regulation of those niches and the possible roles of NCCs in this context is therefore of particular importance.

## Author contributions

CC: Work conception and design, data collection and analysis, manuscript writing. VN: Work conception and design, data collection and analysis, manuscript writing. AG: Revision for intellectual content, final approval of the version to be published. SW: Work conception and design, revision for intellectual content, final approval of the version to be published. BR: Work conception and design, revision for intellectual content, final approval of the version to be published.

### Conflict of interest statement

The authors declare that the research was conducted in the absence of any commercial or financial relationships that could be construed as a potential conflict of interest.

## Abbreviations

BM: bone marrow
BMP: bone morphogenic protein
HSC: hematopoietic stem cell
MSC: mesenchymal stem cell
NCC: neural crest cell
NCSC: neural crest stem cell
NSC: neural stem cell
SVZ: subventricular zone.
